# Incidental pineal gland cyst in girls with early onset of puberty

**DOI:** 10.1186/s13052-022-01235-4

**Published:** 2022-03-21

**Authors:** Gianpaolo De Filippo, Rossella Gaudino, Valeria Calcaterra, Alberto Villani, Elena Bozzola, Mauro Bozzola

**Affiliations:** 1 Assistance Publique-Hôpitaux de Paris, Hôpital Robert-Debré, Service d’Endocrinologie Et Diabétologie Pédiatrique, Paris, France; 2 French Clinical Research Group in Adolescent Medicine and Health, Paris, France; 3 Department of Surgical Sciences, Dentistry, Gynecology and Pediatrics, Pediatric Division, University of Verona, Verona, Italy; 4 Pediatrics and Adolescent Unit, Department of Internal Medicine and Therapeutics, University of Pavia, Pavia, Italy; 5 Pediatric Unit, Vittore Buzzi Children’s Hospital, Milan, Italy; 6 Pediatric Unit, Emergency Department, IRCCS Bambino Gesù Children’s Hospital, Roma, Italy; 7 University of Pavia, Pavia, Italy; 8 Onlus, Il Bambino e il Suo Pediatria, Galliate, Novara, Italy

**Keywords:** Pineal gland, Incidentaloma, Precocious puberty, Central early puberty, Magnetic resonance imaging

## Abstract

**Background:**

The causes of an early onset of puberty are still not clearly defined and may vary from subject to subject. In girls, even if 90% of early puberty is idiopathic, magnetic resonance imaging (MRI) of the brain is performed to exclude secondary causes of precocious puberty, in particular pathological lesions as hypothalamic tumours (hamartoma). In some cases, other intracranial lesions are considered as incidental findings. Aim of the study is evaluating the prevalence of abnormal intracranial lesions detected by brain magnetic resonance imaging MRI with particular focus on the prevalence of pineal gland cysts in the diagnostic work-up of girls with central precocious puberty (CPP) as onset before 8 years and central early puberty (CEP) as onset before 10 years.

**Material and methods:**

MRI data of girls referred from January 2010 to December 2015 to the Pediatric Endocrinology Unit of University of Pavia for early onset of breast development were collected.

**Results:**

We collected 123 MRI data of girls referred to the Pediatric Endocrinology Unit of University of Pavia for early onset of breast development in the study period. Out of them, 25 (20.3%) had cerebral abnormalities and 15 (12.2%) had pineal gland cysts. No significant differences were noted in auxological, ultrasound and hormonal parameters at diagnosis among girls with or without pineal cysts. Patients have been observed for at least three years after the discontinuation of therapy. None of our patients had an unfavorable evolution.

**Conclusions:**

Although pineal cysts seem to be not involved in the onset of puberty, the relevance of the finding remains controversial. Our study wants to provide further insight into the incidence of pineal cysts in pubertal advances. Of note, pineal cysts are often asymptomatic and do not evolve over time.

## Background

Precocious puberty is usually defined as the appearance of breast development before 8 years of age in females and an increase in testicular volume before 9 years in males, associated with an acceleration of height velocity and advanced bone maturation [1]. Moreover, early onset puberty may be observed in girls showing breast development after the ages of 8 but before 10 years. This condition, called central early puberty (CEP) [2], is self-limiting in most girls but may requires careful monitoring as it may rapidly progress to earlier occurrence of the menarche with consequential premature cartilage welding and compromised final stature. Early puberty also has been related to various consequences in the medium and long term such as behavioural problems, obesity, and metabolic comorbidities [3–5].

Precocious puberty may be divided into two main categories based on its aetiology—central precocious puberty (CPP) or gonadotropin-dependent precocious puberty and peripheral precocious puberty (PPP) or gonadotropin-independent precocious puberty. In CPP, the common mechanism of progressive precocious puberty is early activation of pulsatile GnRH secretion, which, in most cases, especially in girls, remains unexplained and thus is defined as idiopathic. However, in the last decade the number of idiopathic cases has declined thanks to the discovery of mutations in different genes including KISS1, KISS1R, MKRN3, and DLK1 that cause CPP [6, 7]. Both females and males with CPP show a significant increase in levels of follicle stimulating hormone (FSH) and, mainly, luteinizing hormone (LH) after gonadotropin-releasing hormone (GnRH) stimulation [1]. With the development of new and more sensitive immunoassays for measuring serum gonadotropins, a single basal serum LH value has been proposed to diagnose CPP [8].

Brain MR imaging (MRI) must be performed to exclude secondary causes of precocious puberty in both sexes, although the prevalence of organic puberty is higher in boys (40–90%) than in girls (8–33%) [1]. After widespread use of MRI in the diagnostic work-up of early onset of puberty, pineal cysts are frequently found and are interpreted as incidentalomas [9].

The aim of this study was to evaluate the prevalence of abnormal intracranial lesions detected by brain MRI in the diagnostic work-up of girls with early onset of puberty with particular focus on the prevalence of pineal gland cysts. Moreover, we tried to establish whether the presence of these intracranial abnormalities may correlate to specific clinic or biological phenotype.

## Materials and methods

This is a retrospective monocentric study.

We recorded MRI data of 123 consecutive girls referred to the Pediatric Endocrinology Unit of University of Pavia, Italy, from January 2010 to December 2015 for early onset puberty. Both the history of the disorder and a physical examination of each girl suggested progressive pubertal development according to the Tanner classification. The patients were followed for at least three years after the end of therapy or completion of the follow up period.

Out of the patients, 100 had a CPP diagnosed according to the classic definition if the appearance of the breast development was before the age of 8 and 23 patients had a CEP defined if the appearance of the breast development was between eight and ten years [2].

For 71 patients it was possible to collect auxological, hormonal and ultrasonographic parameters.

One girl of Italian origin had been adopted; and 8 girls were non-Caucasian (4 South Americans, 3 Africans and 1 Asian).

Girls with dimorphic syndrome, chromosome abnormalities, endocrine or chronic diseases, neurological disorders or CNS pathologies were excluded from the study. None of the girls were overweight or obese, their BMIs being within normal range.

Height was measured using a Harpender stadiometer and expressed as standard deviation score (SDS). Body mass index (BMI) was calculated according to the formula weight (kg)/height^2^ (m^2^). Both height and BMI SDS were calculated according to the charts developed by Cacciari et al. [10] and were within normal range. Bone age was assessed using the Greulich and Pyle Atlas [11].

At time of diagnosis, all patients were evaluated through auxological data including pubertal stages, bone age, basal and peak serum levels of gonadotropins after the GnRH stimulation test, serum estradiol values, and both uterine and ovarian measurements by pelvic ultrasound. All subjects underwent the GnRH stimulation test (100 μg/m^2^ GnRH given i.v. bolus) to evaluate serum luteinizing hormone (LH) and follicle-stimulating hormone (FSH) determined by chemiluminescent immunometric assay (Siemens Medical Solutions Diagnostics, Milan, Italy). We considered stimulated LH levels > 5 IU/l as a diagnostic cut-off of pubertal range.

Thorough pelvic ultrasound using an Aloka Prosound SSD 5500 machine was performed to evaluate uterine length and transverse diameter, fundus/cervix ratio, ovarian volume, and the presence of an endometrial echo. Our study considered as pubertal size a uterine length ≥ 3.5 cm, a fundus/cervix ratio of > 1, an ovarian volume of ≥ 2 ml, and the presence of an endometrial echo.

To identify any hypothalamic or pituitary lesions, all the girls diagnosed with CPP underwent MRI (Siemens MAGNETOM Symphony 1.5 T MRI), using gadolinium-enhanced T1- and T2-weighted MRI to detect occult intracranial lesions. The standardised MR protocol consisted of 3-mm sagittal and coronal T1-weighted spin-echo slices (TR = 400 ms, TE = 115 ms, matrix size = 512 × 512) centred on the pituitary region, 3-mm axial T2-weighted fast spin-echo slices (TR = 5,040 ms, TE = 115 ms, matrix size = 512 × 512) of the entire head, and 1-mm sagittal T2-weighted 3D gradient-echo slices centred on the midline. The standardised MR protocol consisted of 3-mm sagittal and coronal T1-weighted spin-echo slices (TR = 400 ms, TE = 115 ms, matrix size = 512 × 512) centred on the pituitary region, 3-mm axial T2-weighted fast spin-echo slices (TR = 5,040 ms, TE = 115 ms, matrix size = 512 × 512) of the entire head, and 1-mm sagittal T2-weighted 3D gradient-echo slices centred on the midline.

### Ethics approval and consent to participate

All research was carried out in accordance with established Ethical Standards involving human participants. The study was approved by the “*Comitato Etico Area di Pavia*”, the Ethics Committee of the IRCCS Foundation San Matteo Hospital, on 17 May 2016, reference number 20160005680. All participants provided parental written informed consent to use of the data.

### Statistical analysis

Results were expressed as mean ± SD using the SPSS for Windows version 21.0 (IBM SPSS**,** Inc., Chicago, IL, USA). A comparison between the two groups (girls with and without pineal cysts) was performed by Mann–Whitney test. Any association between the variables were found using Spearman correlations. Levels of statistical significance were set at *p* < 0.05.

## Results

MRI identified cerebral abnormalities in 25 (20.3%) of the 123 girls. In particular, 15/123 girls (12%; 60% of those with cerebral abnormalities) had pineal cysts, including 12 isolated, 2 associated with cerebellar tonsil ectopia and one with hypothalamic hamartoma. Two of the isolated cysts were observed in CEP girls whereas the others were found in CPP girls. Therefore, the prevalence of cysts (CPP 13% and CEP 8.7%) was comparable in the two groups, with similar characteristics. None of the girls with pineal cysts exhibited neurological signs at diagnosis or during follow up. Of the girls with early onset puberty, two presented with an occasional non-specific headache; one in particular showed a pineal cyst whereas the other did not. Moreover, other findings were detected by MRI including 6 cysts of pituitary pars intermedia and 3 Rathke cleft cysts in the group of CPP girls, and 1 isolated cerebellar tonsil ectopia in the group of CEP girls. None of these patients exhibited neurological signs at diagnosis or during follow up (data not shown).

No significant differences in auxological, pelvic ultrasound and hormonal parameters were found between girls with or without pineal cysts were found in the subgroup of 71 girls (13 – 18.3% with pineal cyst and 58 – 81.7% without) with complete clinical, biological and ultrasonographic data (Table 1).

**Table 1:** Clinical, biological and ultrasonographic characteristics at diagnosis of girls with and without pineal cysts. Results are expressed as median and IQR

	Girls with pineal cysts *N* 13	Girls without pineal cysts *N* 58	*P* value
Chronological age (years)	*7.0 (6.25–8.45)*	*7.0 (6.00 – 8.00)*	*NS*
Height (SDS)	*0.1 (-0.58 – 1.21)*	*0.64 (-0.11 – 1.34)*	*NS*
Bone age (years)	*8.3 (7.25 – 10.25)*	*8.5 (7.75 – 10.70)*	*NS*
BMI (SDS)	*- 0.39 (-0.90 – 0.37)*	*0.04 (-0.42 – 0.73)*	*NS*
Breast appearance (years)	*6.95 (4.63 – 7.68)*	*7.3 (6.00–8.00)*	*NS*
Uterine length (cm)	*4.0 (3.50 – 4.85)*	*3.9 (3.12–4.80)*	*NS*
Left ovarian volume (ml)	*2.6 (1.12 – 3.80)*	*2.0 (1.34–3.50)*	*NS*
Right ovarian volume (ml)	*2.2 (1.58 – 4.20)*	*(1.65–3.32)*	*NS*
Basal FSH (IU/L)	*2.9 (1.10–3.80)*	*2.7 (1.70–4.30)*	*NS*
Peak FSH (IU/L)	*9.7 (4.75–18.40)*	*9.65 (6.80 -14.25)*	*NS*
Basal LH (IU/L)	*0.3 (0.15–0.75)*	*0.3 (0.15–1.55)*	*NS*
Peak LH (IU/L)	*8.1 (6.30 – 13.70)*	*11.95 (6.82–24.92)*	*NS*
Estradiol (pg/ml)	*22.3 (15.35 – 31.00)*	*22.15 (17.00–28.25)*	*NS*

## Discussion

In this study, we evaluated the brain MRI data of 123 girls with early onset puberty. Of the patients, 20.3% showed cerebral abnormalities and 12.2% had pineal gland cysts. The prevalence of pineal cysts was similar in patients with CPP compared to patients with CEP. None of our patients had an unfavorable evolution of cystic injury at least three years after the end of therapy or completion of the follow up period. In a subgroup of 71 patients, for which clinical, hormonal and ultrasonographic parameters were available, no difference was found between girls with or without pineal cyst.

Most cases of CPP in girls have historically been deemed idiopathic. One of the most stimulating developments in this field has been the identification of genetic mutations underlying sporadic and familial cases of CPP [6, 7]. We recently reported the presence of mutations and polymorphisms of KISS1R and MKRN3 genes, not only in CPP patients but also in subjects with anticipated puberty [12]. The hypothalamic hamartoma represents the most common organic cause in both sexes, usually manifesting before 4 years of age [13]. Hypothalamic hamartoma is almost exclusively found in girls with CPP diagnosed at under 6 years of age as observed in our 2-year-old patient. Moreover, finding a hamartoma supported the diagnosis of CPP but required no treatment other than standard GnRH agonist therapy because of the lack of clinical and MRI evolution. When precocious puberty is caused by a hypothalamic lesion such as a mass or malformation, management of the causal lesion generally has no effect on the course of pubertal development [1]. Hypothalamic hamartomas should not be treated surgically when managing precocious puberty. Surgical treatment is only indicated for large hamartomas with neurological symptoms, such as refractory epilepsy and intracranial hypertension [14]. An alternative diagnosis caused by a different type of hypothalamic lesion should be considered when unexpected enlargement occurs. Since the hypothalamic hamartoma showed no development in size nor neurologic disturbances, no surgical intervention was required in our study patient.

Apart from hypothalamic hamartoma, many central nervous system aberrations predispose to CPP and sometimes may be associated with malignant tumours of the pineal region including choriocarcinoma and germinoma, especially in boys, and very rarely with pineal parenchymal neoplasia such as pineocytoma/pineoblastomas [15]. Most pineocytomas are observed in young adults, but they can be found in childhood [16, 17]. Despite the advances in high-resolution MRI, no definitive radiological methods to distinguish pineal region malignancies containing cystic components from benign pineal cysts have been reported [18]. Indeed, small benign glial cysts of the pineal gland are a common incidental finding in adults, discovered on CT scans and during post-mortem examinations. They are usually less than 5 mm in diameter and, unlike the other lesions mentioned above, they do not give rise to symptoms. On the contrary, large, symptomatic pineal cysts of the same nature have only rarely been described. The most frequent intracranial abnormality found in our group was pineal cyst, found in 12% of girls with early onset of puberty, accounting for 60% of the MRI abnormal findings. The percentage is in agreement with other reports. For example, Lacroix-Boudhriova et al. reported a 10.7% prevalence in a study on precocious puberty, but only girls revealed pineal cysts. Thus the final prevalence in females was 13%, quite similar to our finding [9].

A pineal cyst is a benign affection of the pineal gland on the borderline between a pathological lesion and a variant of normality. Symptoms are often non-specific and vague (for instance, headache, sleep disturbances, vertigo, nausea), which makes it difficult to attribute them to the cyst [19]. According to large magnetic resonance (MR) studies, the prevalence of pineal cysts in the general population is 1–1.5% [20], reaching 2–2.5% in young adults, and it decreases with age. Previous studies regarding the prevalence of asymptomatic pineal cysts have reported occurrence ranging from 1–10% [21]. Pineal cysts are found in other paediatric conditions, suggesting that they are nonspecific for precocious puberty. In fact, no significant differences in the prevalence of cysts within the CPP and idiopathic short stature (ISS) groups have been reported [9].

The clinical approach to pineal cysts is still debated within the neurosurgical community. The majority of patients are asymptomatic and observation is recommended. Microsurgical resection represents an accepted option for benign cysts in children with symptoms including headache, vertigo, numbness, and visual disturbances [22].

In contrast to these incidentalomas, the management of symptomatic pineal cysts is more defined, often requiring a surgical approach involving microsurgical resection and sometimes stereotactic aspiration. Some authors have suggested determining whether the amount of unjustified routine follow-up imaging of incidental pineal cysts in children with no suspicion of neoplasm can be reduced. Gender, cyst size, and shape do not influence the outcome. Because the probability of underlying cystic neoplasm and the growth tendency of pineal cysts is so small, MRI follow-up does not seem to be helpful. Based on these results, they suggested that systematic follow-up of pineal cysts by serial MRI is not indicated in the absence of unusual radiological characteristics or related clinical symptoms [23]. A recent paper on the issue of follow-up of pineal cysts suggests that MRI should be repeated only with cysts > 14 mm and/or with an abnormal radiological pattern or clinical symptoms [24].

Even if MRI is not a part of diagnostic work-out of CEP, in some case it could difficult to differentiate CPP from CEP because the uncertainty on time of thelarche onset. So, we had the opportunity to study a cohort of girls with CEP who underwent cranial MRI.

Although MRI is a helpful technique, it could impose risks on patients due to the use of intravenous contrast agents. Indeed, the US Food and Drug Administration has cautioned against the use of gadolinium-based contrast agents in MRI as gadolinium deposits may remain in the brain for years, especially with the usage of linear gadolinium-based contrast agents rather than macrocyclic agents [25].

## Conclusions

The pineal cyst is a benign affection of the pineal gland on the borderline between a pathological lesion and a variant of normality. The clinical approach to these lesions remains controversial. Pineal cysts are often asymptomatic and do not evolve over time. Routine follow-up including MRI is usually recommended by some authors, even in asymptomatic cases. Moreover, in our study, the lack of differences in auxological, echographic and hormonal parameters between girls with or without pineal cysts and the similar prevalence found in patients with CPP compared to patients with CEP, suggests that intracranial abnormalities detected by MRI do not determine a specific clinical or biological phenotype. Further studies based on large cohorts are necessary to define the ideal interpretation of these benign lesions in patients with early onset of puberty.

## Data Availability

The Authors confirm that the data supporting the findings of this study are available at the authors’office.

## Abbreviations

BMI: Body mass index
CEP: Central early puberty
CPP: Central precocious puberty
FSH: Follicle stimulating hormone
GnRH: Gonadotropin-releasing hormone
ISS: Idiopathic short stature
LH: Luteinizing hormone
MRI: Magnetic resonance imaging
PPP: Peripheral precocious puberty
SDS: Standard deviation score

## References

[1] Carel J-C, Léger J (2008). Precocious Puberty. N Engl J Med.

[2] Paterson WF, McNeill E, Young D, Donaldson MD (2004). Auxological outcome and time to menarche following long-acting goserelin therapy in girls with central precocious or early puberty. Clin Endocrinol (Oxf).

[3] Franceschi R, Gaudino R, Marcolongo A, Gallo MC, Rossi L, Antoniazzi F, Tatò L (2010). Prevalence of polycystic ovary syndrome in young women who had idiopathic central precocious puberty. Fertil Steril.

[4] Murri V, Antoniazzi F, Piazza M, Cavarzere P, Banzato C, Boner A, Gaudino R (2017). Lung Function in Women with Idiopathic Central Precocious Puberty: A Pilot Study. Horm Res Paediatr.

[5] Çoban ÖG, Bedel A, Önder A, Adanır AS, Tuhan H, Parlak M (2021). Psychiatric disorders, peer-victimization, and quality of life in girls with central precocious puberty. J Psychosom Res..

[6] Canton APM, Seraphim CE, Brito VN, Latronico AC (2019). Pioneering studies on monogenic central precocious puberty. Arch Endocrinol Metab.

[7] Simon D, Ba I, Mekhail N, Ecosse E, Paulsen A, Zenaty D, Houang M, Jesuran Perelroizen M, de Filippo GP, Salerno M, Simonin G, Reynaud R, Carel JC, Léger J, de Roux Nl (2016). Mutations in the maternally imprinted gene MKRN3 are common in familial central precocious puberty. Eur J Endocrinol.

[8] Pasternak Y, Friger M, Loewenthal N, Haim A, Hershkovitz E (2012). The utility of basal serum LH in . prediction of central precocious puberty in girls. Eur J Endocrinol.

[9] Lacroix-Boudhrioua V, Linglart A, Ancel PY, Falip C, Bougnères PF, Adamsbaum C (2011). Pineal cysts in children. Insights. Imaging.

[10] Cacciari E, Milani S, Balsamo A (2006). Italian cross-sectional growth charts for height, weight and BMI (2 to 20 yr). J Endocrinol Invest.

[11] Greulich W, Pyle S Radiographic atlas of skeletal development of the hand and wrist. 1959; Stanford University Press.

[12] Pagani S, Calcaterra V, Acquafredda G, Montalbano C, Bozzola E, Ferrara P, Gasparri M, Villani A, Bozzola M (2020). MKRN3 and KISS1R mutations in precocious and early puberty. Ital J Pediatr.

[13] De Brito VN, Latronico AC, Arnhold IJ, Lo LS, Domenice S, Albano MC, Fragoso MC, Mendonca BB (1999). Treatment of gonadotropin dependent precocious puberty due to hypothalamic hamartoma with gonadotropin releasing hormone agonist depot. Arch Dis Child.

[14] Castaño De La Mota C, Martín Del Valle F, Pérez Villena A, Calleja Gero ML, Losada Del Pozo R, Ruiz-Falcó Rojas ML (2012). Hamartoma hipotalámico en la edad pediátrica: Características clínicas, evolución y revisión de la literatura. Neurol.

[15] Kim BS, Kim DK, Park SH (2009). Pineal parenchymal tumor of intermediate differentiation showing malignant progression at relapse: Case Report. Neuropathology.

[16] Stephen M, Zage P, Waguespack S (2011). Gonadotropin-Dependent Precocious Puberty: Neoplastic Causes and Endocrine Considerations. Int J Pediatr Endocrinol..

[17] Osborn AG, Preece MT (2006). Intracranial cysts: Radiologic-pathologic correlation and imaging 8a pproach. Radiology.

[18] Gokce E, Beyhan M (2018). Evaluation of pineal cysts with magnetic resonance imaging. World J Radiol.

[19] Menovsky T, De Ridder D, Grotenhuis JA (2011). Non specific signs related to pineal cysts. Minim Invasive Neurosurg.

[20] Al-Holou WN, Erman SW, Kilburg C, Garton HJ, Muraszko KM, Chandler WF, Ibrahim M, Maher CO (2011). Prevalence and natural history of pineal cysts in adults: Clinical article. J Neurosurg.

[21] Lee DH, Norman D, Newton TH (1987). MR imaging of pineal cysts. J Comput Assist Tomogr.

[22] Choque-Velasquez J, Resendiz-Nieves JC, Rezai Jahromi B, Colasanti R, Raj R, Lopez-Gutierrez K, Tynninen O, Niemelä M, Hernesniemi J (2019). The microsurgical management of benign pineal cysts: Helsinki experience in 60 cases. Surg Neurol Int.

[23] Jussila MP, Olsén P, Salokorpi N, Suo-Palosaari M (2017). Follow-up of pineal cysts in children: is it necessary?. Neuroradiology.

[24] Baldo F (2022). Dealing With Brain MRI Findings in Pediatric Patients With Endocrinological Conditions: Less Is More?. Front Endocrinol.

[25] Radbruch A, Richter H, Fingerhut S, Martin LF, Xia A, Henze N, Paulus W, Sperling M, Karst U, Jeibmann A (2019). Gadolinium Deposition in the Brain in a Large Animal Model: Comparison of Linear and Macrocyclic Gadolinium-Based Contrast Agents. Invest Radiol.

